# Development and pilot testing of an online case-based approach to shared decision making skills training for clinicians

**DOI:** 10.1186/1472-6947-14-95

**Published:** 2014-11-01

**Authors:** Robert J Volk, Navkiran K Shokar, Viola B Leal, Robert J Bulik, Suzanne K Linder, Patricia Dolan Mullen, Richard M Wexler, Gurjeet S Shokar

**Affiliations:** Department of Health Services Research, Unit 1444, The University of Texas MD Anderson Cancer Center, 1515 Holcombe Blvd, Houston, TX 77030 USA; Paul L Foster School of Medicine, Texas Tech University Health Sciences Center at El Paso, 4800 Alberta Ave, El Paso, TX 79905 USA; LPI Global Consultants/Innovative Funding Partners, Prescott, AZ 86305 USA; Division of Rehabilitation Sciences, The University of Texas Medical Branch at Galveston, Galveston, TX 77555 USA; University of Texas School of Public Health, 7000 Fannin, UCT Ste 2522, Houston, TX 77030 USA; Healthwise/Informed Medical Decisions Foundation, 40 Court Street, Suite 300, Boston, MA 02108 USA

**Keywords:** Decision making, Medical education, Primary health care

## Abstract

**Background:**

Although research suggests that patients prefer a shared decision making (SDM) experience when making healthcare decisions, clinicians do not routinely implement SDM into their practice and training programs are needed. Using a novel case-based strategy, we developed and pilot tested an online educational program to promote shared decision making (SDM) by primary care clinicians.

**Methods:**

A three-phased approach was used: 1) development of a conceptual model of the SDM process; 2) development of an online teaching case utilizing the Design A Case (DAC) authoring template, a well-tested process used to create peer-reviewed web-based clinical cases across all levels of healthcare training; and 3) pilot testing of the case. Participants were clinician members affiliated with several primary care research networks across the United States who answered an invitation email. The case used prostate cancer screening as the clinical context and was delivered online. Post-intervention ratings of clinicians’ general knowledge of SDM, knowledge of specific SDM steps, confidence in and intention to perform SDM steps were also collected online.

**Results:**

Seventy-nine clinicians initially volunteered to participate in the study, of which 49 completed the case and provided evaluations. Forty-three clinicians (87.8%) reported the case met all the learning objectives, and 47 (95.9%) indicated the case was relevant for other equipoise decisions. Thirty-one clinicians (63.3%) accessed supplementary information via links provided in the case. After viewing the case, knowledge of SDM was high (over 90% correctly identified the steps in a SDM process). Determining a patient’s preferred role in making the decision (62.5% very confident) and exploring a patient’s values (65.3% very confident) about the decisions were areas where clinician confidence was lowest. More than 70% of the clinicians intended to perform SDM in the future.

**Conclusions:**

A comprehensive model of the SDM process was used to design a case-based approach to teaching SDM skills to primary care clinicians. The case was favorably rated in this pilot study. Clinician skills training for helping patients clarify their values and for assessing patients’ desire for involvement in decision making remain significant challenges and should be a focus of future comparative studies.

**Electronic supplementary material:**

The online version of this article (doi:10.1186/1472-6947-14-95) contains supplementary material, which is available to authorized users.

## Background

Increasing emphasis is being placed on incorporating patients’ values and informed preferences within the clinical decision making process. Shared decision making (SDM) between patients and health care providers plays a prominent role in calls to improve the quality of health care and promoting patient-centered care [1, 2]. Patient centeredness is an essential element of the new Patient-Centered Medical Home model that is widely endorsed and being implemented in the United States as an approach to improving quality [3]. Eliciting patients’ values and preferences have also been advocated as a vital component of the process of evidence-based clinical decision making [4] by the Institute of Medicine (IOM) [5] and by the Patient-Centered Outcomes Research Institute (http://pcori.org). For cancer screening and prevention decisions specifically, shared decision making (SDM) is advocated by authoritative organizations [6].

Although research suggests that patients prefer a SDM approach in healthcare decisions [7] and, at the least, want to be informed about their healthcare decisions [8, 9], physicians do not routinely implement SDM into their practice [10–12]. The IOM goal of a health care system that promotes a fully informed patient participating in SDM with a health care provider remains to be realized [13].

The number of new training programs regarding SDM for health professionals has increased over the past decade [14–16]. Yet, evidence of their effectiveness is generally lacking [15, 17] and provider training around specific behaviors for promoting shared decisions remains a priority [18, 19]. A recent review by Legare et al. [15] identified 54 SDM training programs in 14 countries. Curricula were delivered using a variety of methods, such as small-group sessions, case-based discussion, small-group teaching sessions, audit and feedback, academic detailing, and viewing of videorecorded medical encounters. Heterogeneity in learning objectives, teaching methods, and training and a lack of data on the impact of these training programs on SDM makes it difficult to identify the most effective strategies or components of such programs [15].

In this multi-phased study we utilized a case-based approach to teaching SDM skills to clinicians because this offers some advantages: it incorporates principles of adult learning theory, it is a well-established educational method, it allows direct application of theory to practice [20] and it is thought to be time efficient in conveying a large amount of information in short time frames [21]. This approach is particularly useful for clinicians because it allows direct application of new knowledge to real-life situations that clinicians encounter in everyday clinical practice thus facilitating integration of newly acquired skills into clinical practice. Here we report on the development and pilot testing of an online, clinical case regarding prostate cancer screening to teach primary care clinicians the process of SDM with their patients and skills to integrate it into their practice style.

## Methods

We used a multipronged, sequential process in this project involving three main phases: 1) review of SDM training programs, conceptual models and measurement tools with the goal of creating a comprehensive model of the SDM process to guide development of the case; 2) develop the SDM case using a novel, online case-based approach to medical education; and 3) pilot test the case for acceptability and potential impact on knowledge and confidence in performing SDM.

### Phase 1: a conceptual framework about clinician competencies for SDM

**Figure 1:**
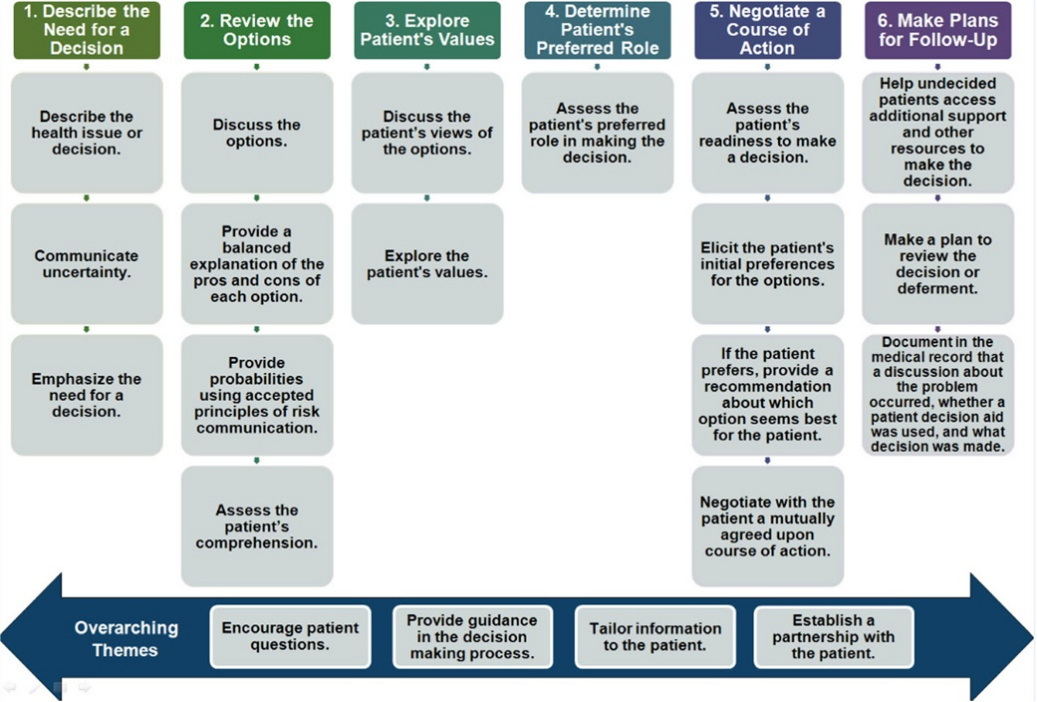
**Conceptual framework for the case: steps in SDM process.**

The conceptual framework for the case came from an extensive review of current SDM training programs, models of informed decision making and SDM, and measurement systems such as questionnaires and interaction coding systems. Our purpose was not to conduct a formal systematic review of SDM training programs. Rather, our goal was to identify the conceptual foundation of existing training programs to provide a starting point for considering a comprehensive approach to SDM training. The resulting six-step process guided the content and structure of the case (Figure 1). SDM competencies are behaviors or performance objectives a clinician should exhibit in promoting an informed decision making process.

#### Identification of sources for clinician SDM competencies

An extensive literature search was performed to identify clinician training programs on SDM. Medline, Scopus, and Cochrane library databases were searched with the limits of English language and a publication date within the last 5 years. Key search terms included: shared decision making, informed decision making, training program, educational program, curriculum, and interventions. No assessment of study quality was done as we were interested in the conceptual basis of the programs and not in the quality of their evaluations. In addition to SDM training programs, we reviewed coding systems and conceptual approaches through our search.

To supplement the literature search, we contacted several leaders in the field of decision aid development and evaluation to ask them to identify additional training programs of which they were aware. We also reviewed presentations from the 2008 Dartmouth Summer Institute on Informed Patient Choice (where the theme was professional education) and abstracts from the 2009 International Shared Decision Making Conference held in Boston. The Additional file 1 contains a listing of the coding systems, conceptual frameworks, and training programs we identified. This list should not be considered exhaustive as we would have missed training programs unknown to our experts or that had not been published.

#### Abstraction of competencies

The training programs, coding systems, and conceptual models were then reviewed by the research team to extract either explicit or implicit competencies clinicians should exhibit in promoting an SDM process. Some of the primary sources were developed to measure patient involvement in physician-patient encounters, so abstracting competencies from these sources was fairly straightforward. Other programs, such as the program developed by O’Connor et al., at the Ottawa Health Research Institute, required our re-expressing instructions for clinicians to reflect the implied competencies. This process resulted in 199 unique competency statements being identified.

#### Grouping competencies into themes and framing them as behaviors

A working group (RJV, PDM, SKL, and VBL) identified overarching themes for the initial list of 199 competencies. Using printed PowerPoint slides of each competency and source, the working group sorted the competencies into 21 broad thematic areas. Approximately 20 of the original 199 competencies were not associated with a specific behavior. Within each of the 21 themes, several competencies were redundant. Similar competencies within each theme were consolidated, which resulted in 62 competencies. These competencies were then used to define the themes as key behaviors a physician should exhibit in promoting an SDM process.

#### Structuring the SDM Process: a conceptual framework

Key behaviors were grouped according to when they might occur during a clinical encounter. Although the decision-making process is not linear, sequencing the behaviors was useful in conceptualizing a model for the SDM process. The final result was six steps for achieving SDM (Figure 1, top row): 1) describe the health issue the patient faces and the need for a decision, 2) discuss with the patient the options (including the pros and cons), discuss the likelihood of important outcomes, and assess the patient’s comprehension, 3) explore what is important to the patient in making a decision, 4) assess the patient’s desired role in making the decision, 5) assess the patient’s readiness to make a decision, assess the patient’s preferences for options, and negotiate a mutually agreed upon course of action, and 6) make plans for follow up and providing support. In considering the behaviors, four in particular were determined to be important throughout the SDM process (Figure 1, bottom row): 1) encouraging patient questions, 2) providing guidance in the decision making process, 3) tailoring information to the patient, and 4) establishing a partnership with the patient. These four are considered overarching behaviors in the model as they are not specific to any particular step.

#### Phase 2: development of the case

For our study, we used Design A Case (DAC, Galveston, TX; http://www.designacase.org/), an innovative, interactive web-based case authoring template, to develop the prostate cancer screening scenario. DAC introduced this case-based approach into the online learning environment, allowing for the development and delivery of online cases for the training of health professionals at all levels. Use of DAC is highly acceptable to learners [22, 23], preferred over other methods of teaching [23], and improves knowledge as assessed on standardized exams [23, 24]. DAC web cases enhance the educational process by taking the learner step by step through an authentic clinical encounter, at his or her own pace, and providing information at the conclusion of each section to aid in the comprehension and application of the material. This new knowledge can then be immediately applied from the virtual patient to the “live” patient in the clinical setting.

DAC is characterized by simulating the clinical setting using a standardized linear format delivered using a case-based learning strategy that promotes critical reasoning skills and reflective thinking. DAC allows the author to efficiently and effectively develop asynchronous interactive web-based cases. DAC has two innovative components, the authoring template which enables the author to develop a web case in approximately 10-12 hours of concentrated time, and the web case repository which houses the web cases. Both components of DAC are login and password protected.

The teaching model for the case was a 60-year-old man presenting to his primary care physician for his annual physical exam, including possibly prostate cancer screening. Development of the case began with storyboarding to make explicit the sequence of learning content and relationships between the learning modules. From there, the authoring template was used to populate the initial case for review and feedback by the team. The case is comprised of a series of modules representing each step of SDM and linking it with the appropriate phase of the clinical encounter. Each module consists of two screens. The first screen contains text that delivers information appropriate to the module topic, which is sometimes augmented with graphics (e.g., photographs) or other multimedia. Below this information, questions are posed, and the learner is required to enter an answer to the questions before accessing the second screen. The second screen contains the “faculty” answer to the question or questions in the first screen, with relevant clinical pearls of wisdom and links to relevant web resources. Decisions were made to include additional DAC features, such as faculty responses to open-ended queries, clinical pearls where specific content can be explored in detail, and links to supplementary resources external to the case.

At this point, a peer review step was added to obtain feedback from experts in SDM and prostate cancer screening who reviewed the entire case online. Additionally, the case was reviewed by a DAC educational expert (GS). Major refinements were suggested from these reviews, including modifications to the presentation format (e.g., larger font, more use of images), moving the SDM steps to the beginning of the case, and adding a module on patient decision aids with links to resource materials. Before we launched the evaluation, the refined case was pilot-tested in a focus group format with primary care physicians to assess the usability and relevance of the case. As a result of the pilot testing, some of the text of the case, clinical pearls, and questions were re-worded for clarity and some new resources (e.g., for health literacy screening) were added.

### Phase 3: pilot testing of the SDM case

#### Participants and recruitment

Study participants were a convenience sample of members of a national primary care research network operated by the American Academy of Family Physicians. An emailed invitation about the project was sent from the network director to academy members with instructions to indicate interest by replying to the study coordinator. Interested members were then given a link to the online case and a unique identifier with a password and instructions on completing the case and evaluation. Several network members forwarded the solicitation to their statewide networks; the same instructions were sent to these interested participants. Upon completion of the case, subjects were directed to an anonymous, 30-question survey. Subjects were compensated $150 in the form of a gift card for their participation. This project was approved by the Institutional Review Board of The University of Texas MD Anderson Cancer Center.

#### Evaluation design, measures, and analysis plan

A post-test only study design was selected for this study due to concerns about sensitizing effects (i.e., asking questions about SDM processes before the intervention might key respondents to attend to these issues more closely and thereby influence their responses to the outcome measures). In addition, assessing the acceptability of the case to the participating clinicians and gaining an indication of its potential impact on SDM behaviors is an appropriate first step in evaluating the SDM training approach.

The evaluation plan was guided by Kirkpatrick’s four levels of learning: reaction, learning, behavior, and results (Table 1) [25]. Data were provided as self-report after completion of the case. Reaction to the program was assessed by reports of case completion, assessment of the learning objectives and educational value of the case, and evaluation of the case structure and features. The learning level was assessed with questions about general SDM knowledge (three questions answered “true” or “false”), recognition of the SDM steps (seven questions answered “yes”, “no”, or “not sure”), and knowledge of patient decision aids (seven questions answered “true” or “false”). To test for response set (i.e., answering questions in a single direction, such as answering “true” to all questions), several knowledge questions were included that reflected a non-SDM orientation (Table 2). Ratings of confidence in performing the SDM behaviors (response options “very”, “somewhat”, and “not confident”) and plans to perform SDM behaviors with patients in the future were also included in the evaluation, although they do not provide a direct assessment of the behavior level (response options using a five-point Likert scale ranged from “much less likely” to “much more likely”). Because there was a single assessment period, the study did not objectively assess the effect of changes in SDM behaviors (i.e., impact level). The results are reported descriptively.

**Table 1:** **Case evaluation plan following Kirkpatrick’s evaluation model**

Level	Description	Proposed strategy
Reaction	Learner’s perception of the curriculum and training program	• Completion of case
		• Assessment of objectives and educational value
		• Structure and features of the case
Learning	Increased knowledge of SDM	• General SDM knowledge
		• Recognition of steps
		• Knowledge of decision aids
Behavior	Transfer of knowledge to practice	• Not directly assessed (ratings were collected about confidence in performing SDM behaviors, and plans to perform SDM behaviors)
Results	Final results that occur because of participation in the program	• Not able to assess

**Table 2:** **Clinician knowledge of SDM and decision aids after completing case**

	Overall (n = 49)	Currently not a decision aid user (n = 31)	Currently a decision aid user (n = 18)
**General Knowledge of SDM**			
SDM is a process between patient and provider in which both parties express values and participate in making a decision.	83.7	87.1	77.8
The clinician alone is best equipped to make the final decision.*	100.0	100.0	100.0
An equipoise decision is one where the scientific evidence does not favor one option over another.	95.6	93.5	100.0
**Knowledge of Steps in SDM Process**			
Describe need for a decision.	95.2	100.0	88.9
Describe options.	100.0	100.0	100.0
Describe one best option to the patient.*	93.9	96.8	88.9
Explore the patient’s values.	100.0	100.0	100.0
Determine the patient’s preferred role.	95.9	96.8	94.4
Negotiate a course of action.	91.8	90.3	94.4
Make plans for follow-up.	100.0	100.0	100.0
**Understanding Purpose of Decision Aids**			
Help people understand their options.	97.9	96.8	100.0
Help people understand the harms and benefits of the options.	98.0	96.8	100.0
Help people think about choices.	98.0	96.8	100.0
Provide information about options.	98.0	96.8	100.0
Help people to deliberate.	77.6	77.4	77.8
Support people to forecast how they might feel.	61.2	64.5	55.6
Help the process of constructing preferences.	85.7	83.9	88.9

*Correct response is false/no.

Although not a perfect way to separate those clinicians who are more favorably inclined to practice SDM, we divided the sample into those clinicians who used patient decision aids in their practice and those who do not. We separated these groups in the analysis as one way to test for selection bias. We previously showed that primary care physicians use a variety of practice styles related to prostate cancer screening, some of which are more consistent with the SDM approach and the use of patient decision aids [26]. We therefore compared the study outcomes for participants who reported they used patient decisions aids in their practices (n = 18) and those who did not (n = 31). Because of the small sample size, we reported differences that were significant at a more liberal *P*-value of <0.10 and did not adjust for the overall type I error rate. Unless otherwise noted, results for the complete cohort (n = 49) are reported.

## Results and discussion

### Clinician characteristics

Seventy-nine clinicians volunteered to participate in the study. Of these, 52 started the case and 50 completed it. One clinician was a pediatrician and was subsequently dropped from the analysis because the clinical context was prostate cancer screening. Forty-nine eligible clinicians reviewed the case and completed the evaluation. The majority were family physicians (39 of 49), 2 were internists, 1 was a nurse practitioner, and the remaining 7 were clinicians from other specialties (including preventive medicine, infectious disease/HIV, and clinical psychology). Twenty-seven practiced in academic centers, 12 were in residency training, and 26 were male. The average length of practice was 17.2 years (range, 4 to 35 years).

### Assessment of the case

Forty-three clinicians (87.8%) reported that the case met all the learning objectives. Forty-three (87.8%) of the clinicians also rated the case’s educational value as very good to excellent, 47 (95.9%) indicated the case was somewhat or highly relevant to their practice, and 47 (95.9%) found the case somewhat to very helpful for other equipoise decisions.

### Ratings of case features

The case was considered well organized by 48 clinicians (98.0%), and all 49 clinicians (100%) felt it provided useful information. Supplementary information accessible via links to information outside of the case was used by 31 clinicians (63.3%), of which 28 (90.3%) found the information useful. Clinicians who did not use decision aids were more likely to rate the case as too long than were clinicians who did use aids (26% versus 6%, *P* = .08).

### Knowledge of SDM and decision aids

General knowledge of SDM after completing the case was excellent (Table 2). In addition, clinicians were able to correctly identify the steps in the SDM process. Knowledge of the purpose of patient decision aids was generally high, although somewhat lower in understanding the functions related to deliberation (77.6% correct) and affective forecasting (61.2% correct).

### Confidence in SDM and intention to perform SDM

**Figure 2:**
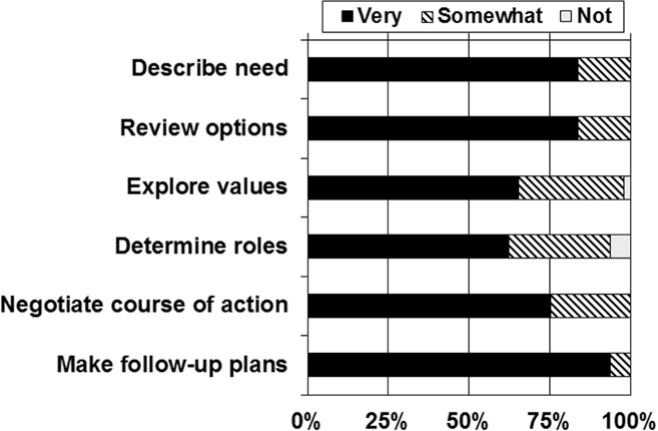
**Confidence in ability to perform steps in SDM process after completing the case (n = 49).**

Overall, 34 (69.4%) of clinicians indicated they felt very confident in their ability to perform SDM with their patients as a result of the case, and the remaining 15 (30.6%) felt somewhat confident. Ratings of confidence varied by the step in the SDM process (Figure 2). While the majority of clinicians felt very confident with each step in the SDM process, confidence was lowest for the steps involving exploring the patient’s values (65.3% very confident) and determining the patient’s preferred role in decision making (62.5% very confident).

After completing this case, more than 70% of clinicians indicated they intended to perform SDM with their patients “often” or “always” (Table 3). Significantly more clinicians who used patient decision aids than those who did not indicated that they planned to perform each of the SDM steps often or always with men facing a prostate cancer screening decision (88.9% versus 64.5%, *P* = .06).

**Table 3:** **Clinicians intentions to perform steps in a SDM process after completing the case**

	Overall (n = 49)	Currently not a decision aid user (n = 31)	Currently a decision aid user (n = 18)
Intention to perform steps in SDM process*			
Describe need for a decision.	87.5	83.3	94.4
Describe options.	89.6	83.3	100.0
Explore the patient’s values.	83.3	73.3	100.0
Determine the patient’s preferred role.	83.3	76.7	94.4
Negotiate a course of action.	93.8	90.0	100.0
Make plans for follow-up.	91.5	89.7	94.4

Numbers represent percentage of correct responses.

*Intention to perform behavior “often” or “always.”

## Discussion

We found a case-based learning approach to SDM skills development guided by a comprehensive model of SDM was well received by primary care clinicians. Clinicians found the structure of the case highly acceptable and the information valuable, and they made use of many of the case’s features. They were able to identify the steps in an SDM process. They further felt confident about the steps in SDM and planned to perform them in the future. They further reported that the case-based approach would be helpful for other equipoise decisions, supporting its use beyond the prostate cancer screening context.

While clinicians generally felt confident about performing SDM with their patients, of note are the areas where additional training might be needed. Clinicians were least comfortable with exploration of their patients’ values about prostate cancer screening and determining how involved their patients wanted to be in making the screening decision. These tasks are decidedly communicative and require a degree of sharing information and co-constructing preferences as part of a patient-centered process.

Keys to encouraging clinician participation in SDM training programs include choice of a clinically relevant topic, interactivity and ease of access, and inclusion of decision support tools in an interesting and professionally stimulating program [27]. Current research does not allow firm conclusions on the most effective interventions for increasing adoption of SDM by clinicians [16]. An attractive feature of our case-based approach is its familiar format and brevity. Some SDM training programs require multiple sessions taught over an extended period of time [28, 29]. For practicing clinicians, an intensive continuing education program may not be feasible. A case-based approach such as the one we developed and tested might be offered as continuing education for clinicians, perhaps qualifying for ethics credit. Yet, it is unlikely that a single infusion of SDM training will result in meaningful and sustained change. Such a program could be part of a broader SDM training and support program, where other established approaches to supporting changes in clinician practice might be implemented [16].

Targeting non-physician health providers for training in SDM skills using case-based approaches may also be an important strategy for implementing SDM [30, 31]. Friedberg et al. [19] in a multisite demonstration project, found the non-physician providers were more enthusiastic about using patient decision aids in clinical practice than physicians were, in part due to having more time to review the tools with patients. The use of health coaches has been investigated as well [31]. In assessing several large randomized trials, Veroff et al. [32] reported that, compared with patients who received usual care, patients with selected preference-sensitive conditions had lower overall healthcare costs when a health coach trained in SDM was part of an enhanced patient support program. It is imperative that successful strategies be developed to implement SDM in clinical care, as SDM is a major national priority for patient-centered outcomes research. New delivery systems, such as a team approach to patient education in patient-centered medical home initiatives (http://www.pcpcc.org/), provide an opportunity to test these approaches in real-world clinical practice.

To our knowledge, this is the first study to use an online case-based approach as the primary strategy for SDM skills training. Other programs have used combinations of in-person workshops, online tutorials, checklists for providers, peer coaching models, and sharing CME materials. (A comprehensive inventory of SDM training programs can be found at http://www.decision.chaire.fmed.ulaval.ca/en/list-of-sdm-programs/). In health professional education, a case-based approach utilizes the concepts of adult learning and offers a form of inquiry-based learning that falls between the structured and guided level that is preferred by learners [33]. This type of learning encourages a deep approach to learning particularly well suited for teaching shared decision making skills to established clinicians. It promotes the understanding of concepts new to the learner [20, 34], links theory directly to practice, and enables organization of the knowledge into the flow of the actual encounter thus encouraging retrieval in a context specific manner [35]. The online delivery of the case also adds convenience, maximizes limited time for education [36], and negates the need for direct involvement by content experts [37]. Furthermore, it enables the learner to tailor their experience to meet their personal learning style by controlling the pace of learning and the content through links they choose to access. Online medical education has become increasingly common and is familiar to practicing clinicians, and evidence shows that web-based approaches in medical education result in high learner satisfaction [38] and result in knowledge gains as high or higher than traditional teaching approaches [39]. Finally, the on-line platform offers a convenient assessment opportunity and CME delivery mechanism that that can aid in wider dissemination of the program.

A strength of this study is the use of a comprehensive model of the SDM process to guide development of the case. The model was drawn from previous conceptual models in the SDM literature, SDM training programs available at the time of the study, and the underlying theoretical models used in various SDM coding systems. We recognize the SDM process does not follow a fixed sequence, and clinicians and patients move between the steps as they make decisions. But, by clarifying the steps in a SDM process were we able to identify those key skills that remain challenging for many clinicians, most notably comfort in exploring patients’ values related to the options and determining how involved patients want to be in making health care choices. These skills are decidedly communicative and should be a primary focus of training programs going forward. Finally, the model includes attention to documentation of the SDM process in the medical record, which may become a requirement for reimbursement.

Our study is limited by recruitment of clinicians affiliated with primary care research networks who are potentially more interested in studies of medical training than other clinicians are. As this was a volunteer sample, the findings may be more favorable than would be expected with other clinician groups due to selection bias. The case was developed and evaluated during the swirling controversy in the medical community about the benefits and harms of prostate cancer screening [40, 41], and the case topic might have distracted from the overall goal of training in SDM processes. Finally, our study addressed the first two levels of Kirkpatrick’s evaluation framework (i.e., reaction and learning) while indirectly considering changes in clinician behavior through self-reported intentions to perform SDM. The design did not include a control or comparator intervention. Additional, comparative research is needed to determine the effect of competency training on clinician-patient interactions and deliberation in promoting informed, shared decisions.

## Conclusions

Barriers to adopting SDM in clinical practice are well known [19] and the lack of clinician training is prominent in this list. We found that the use of an online case-based approach to teaching SDM, grounded in a conceptual model of the SDM steps used in a clinical encounter, led to positive results for at least two of the four of Kirkpatrick’s levels of learning. The approach was highly acceptable, led to acquisition of knowledge and confidence, and increased self-reported intention to practice SDM in the future. The findings of improved knowledge and acceptability are consistent with prior experience with this approach [23, 24].

This study identified two areas where clinicians’ confidence in their SDM skills are lacking, exploring a patient’s values and determining the patient’s preferred role in decision making. These may be new concepts to physicians with little prior experience and training in SDM, so clearly more work is needed in this area. The training content for these steps may be particularly difficult to develop. Developing a video segment to demonstrate these behaviors and integrating it into the case-based approach could improve clinicians’ confidence. With the recent change in the U.S. prostate cancer screening guidelines [40] and the physician and patient concerns that have been raised, the importance of SDM is even more apparent.

## Electronic supplementary material

Additional file 1: **Detailed Summaries of Shared Decision Making Coding Systems, Training Programs, and Conceptual Frameworks Identified for the Project.** (DOCX 54 KB)
