# Supplementary material for: Development and pilot testing of an online case-based approach to shared decision making skills training for clinicians
Source: BMC Med Inform Decis Mak. 2014 Nov 1;14:95. doi: 10.1186/1472-6947-14-95 (PMC4283132; doi:10.1186/1472-6947-14-95)
Supplement: Supplementary file 1 — Additional file 1: Detailed Summaries of Shared Decision Making Coding Systems, Training Programs, and Conceptual Frameworks Identified for the Project. (DOCX 54 KB) [file 12911_2014_855_MOESM1_ESM.docx]

**A Case-Based Approach to Shared Decision Making Skills Training for Clinicians**

**AppendiX**

**Detailed Summaries of Shared Decision Making Coding Systems, Training Programs, and Conceptual Frameworks Identified for the Project**

**Training Programs/INTERVENTIONS**

**Cluster Randomized Trial of Peer Coaching to Increase GP Capacity to Promote Informed Decision Making**

**Developer:** Melina Gattellari, et al.

**What it is:** A randomized controlled trial designed to evaluate whether a peer coaching intervention composed of motivational interviewing approaches and educational materials for general practitioners increases their capacity to promote informed decision making by male patients about PSA screening.

**Use:** The trial used several measures to evaluate GP knowledge, perceptions of patient involvement in informed decision making, GPs’ own decisional conflict, and perceptions of medicolegal risk. The measures the developers used to asses “skills to promote informed decision making by men” were used [by our group] to abstract processes the intervention developers thought important to occur in order to achieve shared decision making.

**Method:** Developers selected content for the intervention based on systematic reviews of the literature, empirical studies, results of prior research by the developers, and Cochrane reviews.

**Articles/References:**

Gattellari M, Donnelly N, Taylor N, Meerkin M, Hirst G, Ward JE, et al. Does 'peer coaching' increase GP capacity to promote informed decision making about PSA screening? A cluster randomised trial. Fam Pract. 2005;22(3):253-65. PubMed PMID: 15824055.

**SIP23 - Promoting Informed Patient Decision Making about Prostate Cancer Screening: Making it Work in Clinical Practice**

**Developer:** Patricia Dolan Mullen and Robert J. Volk

**What it is:** A continuing medical education program designed to teach physicians about informed decision making and facilitate informed decision making for prostate cancer in clinical practice.

**Use:** The continuing medical education program was used as part of cluster randomized trial to determine the effectiveness of provider interventions to promote IDM for prostate cancer screening. The measures the developers used to assess key behaviors for informed decision making were used to abstract processes important for informed decision making.

**Method:** Developers selected content for the intervention based on the work of Gattellari et al. on peer coaching to increased informed decision making for prostate cancer screening, work on guiding clinicians in brief interventions for tobacco cessation, feedback from Gary Kreps, Evelyn Chan and Clarence Braddock, and local pre-testing with physicians.

**Articles/References:**

Mullen PD, Volk RJ, Kneuper S, Chan E, Wuelling S, Spann SJ. Defining and implementing informed decision making (IDM) for prostate cancer screening for the primary care physician. Abstract presented at: 32nd American Society for Preventive Oncology Annual Meeting; 2008 March 17; Bethesda, MD.

**Do Continuing Medical Education (CME) Articles Foster Shared Decision Making?**

**Developer:** Michel Labrecque

**What it is:** A presentation and manuscript sharing results of a study examining whether CME articles on therapeutic or preventive interventions provide evidence-based information on benefits and harms of available options.

**Use:** The measures the developers chose were used to abstract processes important for informed decision making.

**Method:** Developers used a 10-item checklist based on the first two sections of the International patient Decision Aids Standards (IPDAS) to gauge whether CME articles provided enough information on benefits and harms of options.

**Articles/References:**

Labrecque M. Do Continuing Medical Education (CME) Articles Foster Shared Decision Making? Presented at: International Shared Decision-Making Conference; 2009 June; Boston, MA.

Labrecque M, Lafortune V, Lajeunesse J, Lambert-Perrault AM, Manrique H, Blais J, et al. Do continuing medical education articles foster shared decision making? J Contin Educ Health Prof. 2010;30(1):44-50.

**DECISION+: Training Providers in SDM Regarding the Use of Antibiotics for Acute Respiratory Infections**

**Developer:** Michel Labrecque and France Légaré

**What it is:** A presentation and article sharing results of a evaluating a continuing professional development intervention for shared decision making about use of antibiotics for respiratory infections.

**Use:** The key components of the conceptual framework were used to abstract processes important for informed decision making.

**Method:** Developers designed three 180 minute workshops designed to address key components of the conceptual framework created by the developers: estimating the diagnostic probabilities, available evidence on the balance of risk and benefits, and active participation of patients in decision making.

**Articles/References:**

Labrecque M. & Légaré F. Decision PLUS: Training Providers in SDM Regarding the Use of Antibiotics for Acute Respiratory Infections. Presented at: International Shared Decision-Making Conference; 2009 June; Boston, MA.

Légaré F, Labrecque M, LeBlanc A, Thivierge R, Godin G, Laurier C, et al. Does training family physicians in shared decision making promote optimal use of antibiotics for acute respiratory infections? Study protocol of a pilot clustered randomised controlled trial. BMC Fam Pract. 2007;8.

**When Making a Decision is Difficult: Training Workshop**

**Developer:** France Légaré, et al.

**What it is:** A participant’s handbook for a workshop consisting of videos and group discussions about decision support.

**Use:** The workshop is designed to help clinicians recognize situations when a patient is apt to experience difficulty making a decision; identify a patient facing a difficult decision, identify modifiable factors that contribute to the decision, and recognize the barriers and facilitating factors in integrating this new knowledge into practice. The steps for supporting someone making a difficult decision were used to abstract competencies for SDM.

**Method:** Information on the development of the workshop was not available, but the training guide is structured around the Ottawa Decision Support Framework.

**Articles/References:**

Légaré, F., Cote, L., Borduas, F., O'Connor, A., & Saucier, D. When Making a Decision is Difficult: Training Workshop, Participant’s Handbook. Retrieved 2009 October 23, from <http://decision.chaire.fmed.ulaval.ca/index.php?id=93&L=2>.

**Ottawa Decision Support Tutorial**

**Developer:** Annette O’Connor & Mary Jane Jacobsen

**What it is:** A tutorial for practitioners to develop skills in decision support. An online tutorial is available as well as a downloadable PDF guide.

**Use:** The tutorial is designed to help clinicians recognize decisional conflict and contributing factors, describe concepts of decision support, tailor decision support to clients’ needs, use decision support tools and identify skills for improving communication. Competencies for SDM were abstracted from the text of the tutorial on decision support skills.

**Method:** Information on the development of the tutorial was not available, but the tutorial is structured around the Ottawa Decision Support Framework.

**Articles/References:**

O'Connor A, Stacey D, & Jacobsen MJ. Ottawa Decision Support Tutorial. 2007. Retrieved 2009 October 15, from [https://decisionaid.ohri.ca/ODST/odst.php.](https://decisionaid.ohri.ca/ODST/odst.php)

O'Connor A, & Jacobsen MJ. Decisional Conflict: Supporting People Experiencing Uncertainty about Options Affecting their Health. 2007. Retrieved 2009 October 15, [https://decisionaid.ohri.ca/ODST/pdfs/ODST.pdf.](https://decisionaid.ohri.ca/ODST/pdfs/ODST.pdf)

**Supporting Patients Facing Difficult Health Care Decisions: Use of the Ottawa Decision Support Framework (OSDF)**

**Developer:** France Légaré

**What it is:** A decision support intervention for physicians consisting of feedback from participants, a reminder at the point of care, and an interactive workshop including videos.

**Use:** The intervention is designed to increase use of decision support strategies by physicians. Competencies for SDM were abstracted from the actions physicians can take to provide decision support as part of the OSDF process and tools.

**Method:** The decision support intervention tested was based on principles of the Ottawa Decision Support Framework.

**Articles/References:**

Légaré F, O'Connor AC, Graham I, Saucier D, Côté L, Cauchon M, et al. Supporting patients facing difficult health care decisions: Use of the Ottawa Decision Support Framework. Canadian Family Physician. 2006;52(4):476-7.

**How Do We Teach SDM Communication Skills to Clinicians?**

**Developer:** Nan Cochran

**What it is:** A presentation on SDM communication behaviors of clinicians

**Use:** The presentation is meant to convey the communication skills clinicians need to perform shared decision making with patients, highlight barriers to SDM, and highlight the skills physicians most need to improve.

**Method:** The communication skills presented are a synthesis of work by France Légaré, Glyn Elwyn, and Charles Braddock along with details of the risk communication skills necessary for engaging in shared decision making.

**Articles/References:**

Cochran N. How Do We Teach SDM Communication Skills to Clinicians? Presented to the Foundation for Informed Medical Decision Making; 2009 September 10; Boston, MA. For updated presentation slides, contact the author.

**Medical Education in Shared Decision-Making: A Train-the-Trainer Approach to Enhance Physicians’ SDM-specific Communication Skills**

**Developer:** Martin Harter, et al.

**What it is:** A presentation on teaching shared decision making using a train-the-trainer approach.

**Use:** The presentation is meant to portray the basics of SDM, methods and outcomes of SDM training for health professionals and patients, necessary competencies to teach SDM, and didactic methods for SDM trainings in different settings.

**Method:** The SDM skills presented are based on a questionnaire developed to assess the process of SDM from the patient’s perspective. Items for that questionnaire were developed from a model of shared decision making based on the work of Glyn Elwyn, additional theories from general psychology, social psychology and decision analysis. The developers then used a Delphi process to reach consensus on the sequence of steps needed for shared decision making, resulting in a 9 sequential steps for SDM.

**Articles/References:**

Harter M, Loh A, & Buchholz A. Medical Education in Shared Decision-Making: A Train-the-Trainer Approach to Enhance Physicians’ SDM-specific Communication Skills. Presented at: International Shared Decision-Making Conference; 2009 June; Boston, MA.

Simon D, Schorr G, Wirtz M, Vodermaier A, Caspari C, Neuner B, et al. Development and first validation of the shared decision-making questionnaire (SDM-Q). Patient Educ Couns. 2006;63(3):319-27. doi: 10.1016/j.pec.2006.04.012. PubMed PMID: 16872793.

**Interprofessional Education About ISDM in Post-Licensure Continuing Education**

**Developer:** Bettina Berger, et al.

**What it is:** A presentation on interprofessional education about informed shared decision making.

**Use:** The presentation is meant promote interprofessional education about informed shared decisionmaking to engage patients in SDM.

**Method:** The SDM skills presented are based on the work of Towle, with the addition of one competency: eliciting, acknowledging and managing patients’ emotions around decisions.

**Articles/References:**

Berger B, Bieber C, Kelly C, Kryworuchko J, Lillie D, Loh A, Lown B. Interprofessional education about ISDM in post-licensure continuing education. Presented at: Summer Institute for Informed Patient Choice; 2008 June; Hanover, NH.

**The SCOPED Process for Guiding People to Good Decisions**

**Developer:** Jeff Belkora

**What it is:** A presentation on the SCOPED checklist, a guide for clinicians to assist with planning consultation with patients.

**Use:** The presentation is meant promote the use of the SCOPED checklist to promote informed medical decision making in patient consultations.

**Method:** No information was provided in the presentation regarding the development of the SCOPED checklist.

**Articles/References:**

Belkora, J. The SCOPED Process for Guiding People to Good Decisions. Presented at: International Shared Decision-Making Conference; 2009 June; Boston, MA.

**Coding Schemes**

**The Informed Decision-Making Scale**

**Developer:** Clarence Braddock, et al.

**What it is:** A coding tool based on the informed decision-making model composed of nine elements scored from 0 to 2 (IDM-18). The nine elements cover two broad dimensions, providing relevant information and fostering patient involvement.

**Use:** Intended for coding the presence or absence of elements of informed decision making from audio recordings of patient-provider interactions.

**Method:** Developers derived elements of effective communication in the decision-making process from literature on patient-physician communication, informed consent and shared decision making.

**Articles/References:**

Braddock C, III, Hudak PL, Feldman JJ, Bereknyei S, Frankel RM, Levinson W. "Surgery is certainly one good option": quality and time-efficiency of informed decision-making in surgery. J Bone Joint Surg Am. 2008;90(9):1830-8. Epub 2008/09/03. doi: 90/9/1830 [pii]. 10.2106/JBJS.G.00840. PubMed PMID: 18762641; PubMed Central PMCID: PMC2657309.

Braddock CH, 3rd, Edwards KA, Hasenberg NM, Laidley TL, Levinson W. Informed decision making in outpatient practice: time to get back to basics. JAMA. 1999;282(24):2313-20. Epub 1999/12/28. doi: joc91394 [pii]. PubMed PMID: 10612318.

**The OPTION (Observing Patient Involvement) Scale**

**Developer:** Glyn Elwyn, et al.

**What it is:** An instrument designed to assess the extent to which practitioners involve patients in decision making processes.

**Use:** Intended for scoring the extent to which clinicians involve patients in the decision making process by reviewing audio or video recordings of consultations.

**Method:** Developers formulated scale items based on findings of a systematic review of measures of patient involvement and a skills framework of competences for involving patients in healthcare choices.

**Articles/References:**

Elwyn G, Edwards A, Wensing M, Hood K, Atwell C, Grol R. Shared decision making: developing the OPTION scale for measuring patient involvement. Qual Saf Health Care. 2003;12(2):93-9. Epub 2003/04/08. PubMed PMID: 12679504; PubMed Central PMCID: PMC1743691.

Elwyn G, Edwards A, Kinnersley P, Grol R. Shared decision making and the concept of equipoise: the competences of involving patients in healthcare choices. Br J Gen Pract. 2000;50(460):892-9. Epub 2001/01/06. PubMed PMID: 11141876; PubMed Central PMCID: PMC1313854.

Elwyn G, Edwards A, Mowle S, Wensing M, Wilkinson C, Kinnersley P, et al. Measuring the involvement of patients in shared decision-making: a systematic review of instruments. Patient Educ Couns. 2001;43(1):5-22. Epub 2001/04/20. doi: S073839910000149X [pii]. PubMed PMID: 11311834.

**Decision Analysis System for Oncology (DAS-O)**

**Developer:** R. F. Brown, et al. (Medical Psychology Research Unit at the University of Sydney)

**What it is:** A system for coding shared decision making in the oncology setting. The DAS-O consists of two subscales: establishing an SDM framework and providing clear and unbiased information

**Use:** Intended for scoring audio-taped interactions between clinicians and patients for the presence or absence and quality of key components of SDM.

**Method:** Developers formulated the coding system using Braddock’s framework of informed decision making and the Charles et al. shared treatment decision-making model, and original research analyzing audio-taped conversations form an ethical linguistic and psychological perspective on shared decision making.

**Articles/References:**

Brown RF, Butow PN, Ellis P, Boyle F, Tattersall MH. Seeking informed consent to cancer clinical trials: describing current practice. Soc Sci Med. 2004;58(12):2445-57. Epub 2004/04/15. doi: 10.1016/j.socscimed.2003.09.007S0277953603004714 [pii]. PubMed PMID: 15081196.

Butow P, Juraskova I, Chang S, Lopez AL, Brown R, Bernhard J. Shared decision making coding systems: How do they compare in the oncology context? Patient Educ Couns. 2009. Epub 2009/08/04. doi: S0738-3991(09)00251-1 [pii]. 10.1016/j.pec.2009.06.009. PubMed PMID: 19647966.

**Decision Support Analysis Tool (DSAT)**

**Developer:** Pierrette Guimond, et al. (University of Ottawa)

**What it is:** A coding system designed to assess the presence or absence of 22 behaviors in six categories of decision support skills and four categories of communication skills.

**Use:** Intended for evaluating practitioner knowledge of decision support skills in educational settings.

**Method:** Developers based the measure on the Ottawa Decision Support Framework, which incorporates theories of decisional conflict, social support, and expected utility, and Ivey’s problem-solving model.

**Articles/References:**

Guimond P, Bunn H, O'Connor AM, Jacobsen MJ, Tait VK, Drake ER, et al. Validation of a tool to assess health practitioners' decision support and communication skills. Patient Educ Couns. 2003;50(3):235-45. Epub 2003/08/06. doi: S0738399103000430 [pii]. PubMed PMID: 12900093.

Butow P, Juraskova I, Chang S, Lopez AL, Brown R, Bernhard J. Shared decision making coding systems: How do they compare in the oncology context? Patient Educ Couns. 2009. Epub 2009/08/04. doi: S0738-3991(09)00251-1 [pii]. 10.1016/j.pec.2009.06.009. PubMed PMID: 19647966.

**Conceptual Frameworks**

**U.S. Preventive Services Task Force Definition of Informed Decision Making**

**Developer:** U.S. Preventive Services Task Force

**What it is:** A working definition of what constitutes informed decision making and shared decision making.

**Use:** Designed to guide a review on promoting IDM about cancer screening in communities and healthcare systems.

**Method:** The review team consulted the work of others in IDM literature (O’Connor, Elwyn, Wolf, Entwhistle, Rimer, Sheridan, Raiffer, etc.), literature on decision analysis and risk communication, and the USPSTF.

**Articles/References:**

Briss P, Rimer B, Reilley B, Coates RC, Lee NC, Mullen P, et al. Promoting informed decisions about cancer screening in communities and healthcare systems. Am J Prev Med. 2004;26(1):67-80. Epub 2004/01/01. doi: S0749379703002885 [pii]. PubMed PMID: 14700715.

**The Shared Treatment Decision-Making Model**

**Developer:** Cathy Charles, et al.

**What it is:** A conceptual framework of shared treatment decision-making that identifies different analytic steps in the treatment decision-making process, recognizes the dynamic nature of the process, and identifies approaches that lie between the paternalistic, shared, and informed models.

**Use:** Intended to clarify the concept of shared treatment decision-making and to show how this approach fits in the spectrum of models of decision making that range from paternalistic to informed.

**Method:** Based on research by the developers on the meaning of shared decision making to women with early stage breast cancer and further analytic thinking in refining an earlier model by the developers.

**Articles/References:**

Charles C, Gafni A, Whelan T. Decision-making in the physician-patient encounter: revisiting the shared treatment decision-making model. Soc Sci Med. 1999;49(5):651-61. Epub 1999/08/19. doi: S0277953699001458 [pii]. PubMed PMID: 10452420.

Charles C, Gafni A, Whelan T. Shared decision-making in the medical encounter: What does it mean? (Or it takes at least two to tango). Social Science & Medicine. 1997;44(5):681-92. PubMed PMID: ISI:A1997WF20500013.

**Competencies for Informed and Shared Decision Making (ISDM): what the doctor should be able to do**

**Developer:** Angela Towle and William Godolphin

**What it is:** A set of necessary and sufficient competencies for capacity to engage in informed shared decision making.

**Use:** The competencies are intended to guide teaching, learning, practice, and research on informed shared decision making.

**Method:** Developers identified a draft list of competencies from literature searches using electronic databases and references listed in textbooks. The developers then tested the validity of the competencies identified in semi-structured interviews with family doctors, patients, and patient educators.

**Articles/References:**

Towle A, Godolphin W. Framework for teaching and learning informed shared decision making. BMJ. 1999;319(7212):766-71. Epub 1999/09/17. PubMed PMID: 10488010; PubMed Central PMCID: PMC1116602.

Towle A, Godolphin W, Grams G, Lamarre A. Putting informed and shared decision making into practice. Health Expect. 2006;9(4):321-32. PubMed PMID: 17083559.

**Integrative Model of Shared Decision Making in Medical Encounters**

**Developer:** Gregory Makoul and Marla L. Clayman

**What it is:** A definition of shared decision making that integrates the existing literature and outlines essential elements for SDM.

**Use:** The integrative definition of SDM is intended to guide further research in SDM by focusing the description and operationalization of SDM.

**Method:** Developers performed a systematic review of the literature to identify articles in English that included a conceptual definition of shared decision making in the provider-patient context. Elements of SDM in the definitions were then coded into mutually exclusive categories in two groups: behaviors needed to achieve SDM and general qualities of SDM. The authors used elements appearing in at least ten percent of the definitions along with two elements the authors thought important to SDM to create an integrative model of SDM consisting of essential elements, ideal elements and general qualities of SDM.

**Articles/References:**

Makoul G, Clayman ML. An integrative model of shared decision making in medical encounters. Patient Educ Couns. 2006;60(3):301-12. PubMed PMID: 16051459.
